# Extracorporeal Membrane Oxygenation Outside the Operating Room in Neonates and Infants

**DOI:** 10.1093/icvts/ivag235

**Published:** 2026-08-25

**Authors:** Keisuke Shibagaki, Yuji Tominaga, Masashi Takeshita, Takuji Watanabe, Masaya Nakamizo, Hiroyuki Kamiya, Shigemitsu Iwai

**Affiliations:** Department of Pediatric Cardiovascular Surgery, National Cerebral and Cardiovascular Center, Suita, Osaka 564-8565, Japan; Department of Cardiac Surgery, Asahikawa Medical University, Asahikawa, Hokkaido 078-8510, Japan; Department of Pediatric Cardiovascular Surgery, National Cerebral and Cardiovascular Center, Suita, Osaka 564-8565, Japan; Department of Pediatric Cardiovascular Surgery, National Cerebral and Cardiovascular Center, Suita, Osaka 564-8565, Japan; Department of Pediatric Cardiovascular Surgery, National Cerebral and Cardiovascular Center, Suita, Osaka 564-8565, Japan; Department of Pediatric Cardiovascular Surgery, National Cerebral and Cardiovascular Center, Suita, Osaka 564-8565, Japan; Department of Cardiac Surgery, Asahikawa Medical University, Asahikawa, Hokkaido 078-8510, Japan; Department of Pediatric Cardiovascular Surgery, National Cerebral and Cardiovascular Center, Suita, Osaka 564-8565, Japan

**Keywords:** extracorporeal cardiopulmonary resuscitation, neurological outcomes, bleeding

## Abstract

**Objectives:**

Long-term outcomes of emergent extracorporeal membrane oxygenation (ECMO) initiated outside the operating room remain poorly defined. We evaluated long-term outcomes after emergent ECMO support outside the operating room in neonates and infants.

**Methods:**

We retrospectively evaluated 59 neonates and infants requiring emergent ECMO support outside the operating room at our institution (2010-2024). The primary outcome was overall survival after ECMO support. Secondary outcomes included in-hospital outcomes, mortality risk factors, and the impact of manual cardiopulmonary resuscitation (CPR) duration at ECMO cannulation.

**Results:**

Median age and body weight at emergent ECMO were 1.6 months and 3.2 kg, respectively. Indications for ECMO were extracorporeal CPR in 40 (68%) patients, cardiogenic shock in 12 (20%), septic shock in 1 (2%), and respiratory failure in 6 (10%). The median manual CPR duration was 36 min. A CPR duration exceeding 36 min was associated with cervical ECMO cannulation (*P* = .001) and hypoxic encephalopathy (*P* = .008). The median ECMO duration was 4 days. Forty-two (71%) patients were weaned from ECMO, and 32 (54%) survived to discharge. At a median follow-up of 3.7 years, overall survival after ECMO support was 57%, 47%, and 44% at 1, 3, and 5 years, respectively. Multivariable analysis detected non-cerebral bleeding (*P* = .049) and lack of catheter/surgical intervention (*P* = .004) as mortality risk factors.

**Conclusions:**

Long-term survival after emergent ECMO outside the operating room was acceptable in neonates and infants. Strategies to reduce bleeding complications and minimize resuscitation time may further improve outcomes.

**Clinical trial registration number:**

R19092; December 20, 2020

## Graphical abstract

**Figure:**
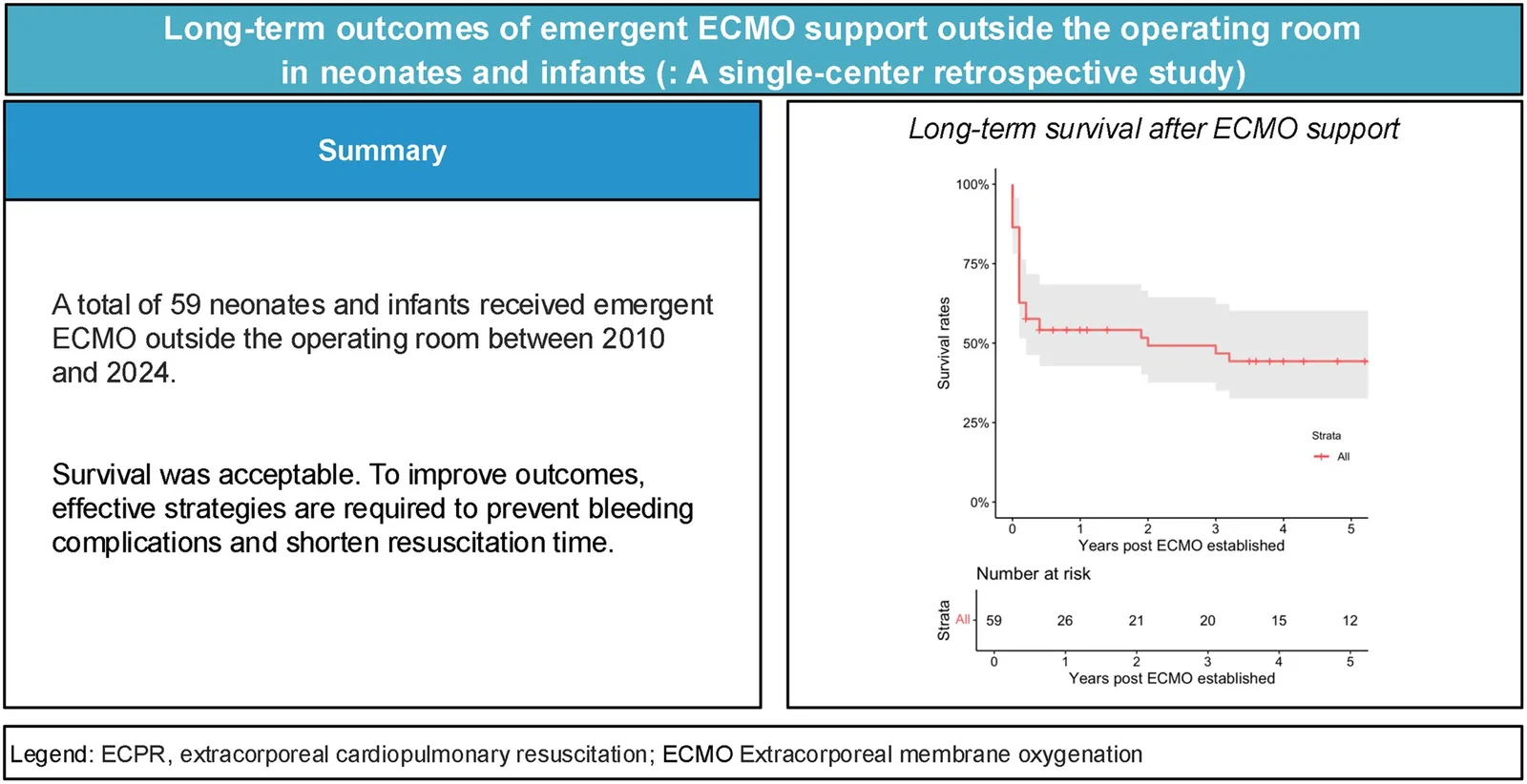

## INTRODUCTION

In the field of paediatric heart disease, extracorporeal membrane oxygenation (ECMO) is commonly used as the primary treatment for cardiopulmonary collapse in children.^1–3^ Extracorporeal membrane oxygenation support is required both for patients who fail to be weaned off cardiopulmonary bypass during cardiovascular surgery and for those who develop cardiopulmonary collapse outside the operating room. In these patients, ECMO is often initiated emergently because of refractory cardiac arrest or impending circulatory or respiratory collapse despite maximal medical therapy.

Although outcomes of paediatric ECMO support have improved in recent years,^1^^,^^4^ patients requiring emergent ECMO outside the operating room continue to experience substantial mortality and morbidity. Several studies have reported that survival-to-discharge rates range from 33% to 66% among paediatric patients requiring extracorporeal cardiopulmonary resuscitation (CPR).^3^^,^^5–8^ Moreover, mortality and morbidity rates remain high in neonates and infants. Analysis of the Extracorporeal Life Support Organization registry revealed an estimated overall survival-to-discharge rate of 44% for neonatal emergent ECMO support.^9^

Given the paucity of data on long-term survival and neurological outcomes in this population, this study aimed to evaluate the long-term outcomes of emergent ECMO support performed outside the operating room in neonates and infants. We hypothesized that acceptable long-term outcomes could be achieved despite the high-risk nature of emergent ECMO and that specific factors would be associated with mortality.

## METHODS

### Ethical approval

This study was approved by the ethics committee of our hospital on January 21, 2020 (approval number: 19092) and conducted in accordance with the ethical standards of the Helsinki Declaration. Given the retrospective, observational nature of the study design, written informed consent was obtained from all participants or their parents/guardians in cases involving minors. Clinical data were retrospectively collected from medical records and recorded in an institutional ECMO database under ethics committee oversight. Biological specimens are stored in the institutional biobank only from participants who provide separate informed consent.

### Patients

Between 2010 and 2024, 59 neonates and infants required in-hospital emergent ECMO support outside the operating room at our institution. Neonates were defined as patients aged <28 days and infants as patients aged <1 year. Extracorporeal membrane oxygenation was initiated in patients who experienced cardiopulmonary arrest without return of spontaneous circulation and in those with impending cardiopulmonary collapse despite maximal medical therapy. Therefore, the study population also included patients who received veno-venous ECMO support. At our institution, ECMO is generally considered in patients receiving high-dose catecholamine support who develop refractory shock with anuria and persistent hypotension or progressive respiratory failure judged likely to result in cardiopulmonary collapse. Patients who required ECMO after cardiac surgery in the operating room or as a bridge to left ventricular assist device implantation were excluded because the objective of this study was to evaluate unplanned emergent ECMO support outside the operating room.

### Management of ECMO

Extracorporeal membrane oxygenation was preferentially established via central cannulation in patients who had undergone cardiac surgery within the preceding 1-2 weeks or weighed less than 2500 g. In all other patients, ECMO was established via cervical cannulation. During ECMO support, all patients were deeply sedated with muscle relaxants. Daily cranial echography was performed for early detection of cranial Haemorrhage. Centrifugal pump flow was maintained at ≥120 mL/kg/min depending on anatomical properties, with a target mean blood pressure of 40 mm Hg in neonates and 50 mm Hg in infants. Systemic vasodilators were aggressively administered for better peripheral perfusion. Diuretics were routinely administered to manage fluid overload, and peritoneal dialysis and extracorporeal ultrafiltration were used as renal replacement therapy when clinically indicated. At our institution, renal replacement therapy was initiated in patients with persistent oliguria (<0.5 mL/kg/hour for 6-12 hours), furosemide-resistant fluid overload (including pleural effusion or ascites), increasing serum creatinine levels, or clinically significant electrolyte or acid-base abnormalities. For anticoagulation therapy, continuous heparin administration was maintained to achieve an activated clotting time (ACT) of 160-200 s or an activated partial thromboplastin time (APTT) of 50-70 s. Platelets and fresh thawed plasma were transfused to maintain the platelet count above 100 000/mm^3^, and the prothrombin time–international normalized ratio was maintained at <1.5.

### Outcomes

The primary outcome was overall survival after emergent ECMO support, assessed using the Kaplan–Meier method. The secondary outcomes included in-hospital outcomes, causes of death and complications, and risk factors for mortality, assessed by multivariable analysis. Additionally, we performed a secondary analysis of patients who required manual CPR at the time of ECMO cannulation, evaluating patient characteristics and complications according to CPR duration, as well as outcomes in patients with hypoxic ischaemic encephalopathy (HIE).

### Statistical analyses

All statistical analyses were performed using SPSS version 31.0 (IBM Corp). Continuous variables are presented as medians with IQRs, and categorical variables are presented as numbers or percentages. Comparisons between the groups were performed using the Mann–Whitney *U* test for continuous variables and Fisher’s exact test for categorical variables, as appropriate. The extent of missing data was assessed for all study variables (**Table S1**). Only body surface area (1.7%) and CPR duration (3.4%) had missing values; all other variables were complete. Given the minimal extent of missing data, no imputation was performed, and analyses were conducted using complete-case data. Survival was estimated through Kaplan–Meier methods. Cox regression analysis was performed to identify the risk factors associated with mortality. Variables with a *P*-value of <.005 in the univariate analysis were included in the multivariable analysis. Hazard ratios (HRs) and 95% CIs were calculated for each variable. The proportional hazards assumption was evaluated using Schoenfeld residuals. Statistical significance was set at *P* < .05.

## RESULTS

### Patient characteristics

The baseline characteristics of the 59 patients are summarized in **Table 1**. The median age and body weight of patients at ECMO cannulation were 1.6 (IQR 0.5-4.3) months and 3.2 (2.6-4.1) kg, respectively. Eighteen (31%) patients were neonates.

**Table 1.** Preoperative Patient Characteristics

Variable	Value (*n* = 59)
Age, months	1.6 (0.5-4.3)
Age <1month	18 (31)
Body weight, kg	3.2 (2.6-4.1)
BSA, m^2^	0.20 (0.18-0.23)
Female sex	33 (56)
Single ventricle	38 (64)
Asplenia	6 (10)
Fundamental diagnosis	
Congenital heart disease	54 (92)
HLHS/HLHS variant	20 (34)
TAPVC	3 (5)
TGA	3 (5)
Ebstein	5 (9)
Myocarditis/Cardiomyopathy	4 (7)
Atrial tachycardia	1 (2)
Postoperative cardiac surgery	44 (75)
Postoperative period, day	7 (1-19)
Within 24 hours	16 (27)
24-72 hours	3 (5)
72 hours to 1 week	7 (12)
More than 1 week	20 (34)
Indication for ECMO	
ECPR	40(68)
Cardiogenic shock	12 (20)
LOS	7 (12)
Tachycardiac arrhythmia	2 (3)
Coronary trouble	1 (2)
Fontan failure	1 (2)
Myocarditis	1 (2)
Septic shock	1 (2)
Respiratory	6 (10)
CPR time, min	36 (23-51)
ECMO procedure	
ECMO duration, days	4 (2-8)
Location	
Intensive care unit	57 (97)
Cardiac catheterization laboratory	2 (3)
Cannulation site	
Central	39 (67)
Cervical	20 (33)
System	
Veno-arterial	55 (93)
Veno-venous	4 (7)
Intervention on ECMO	23 (39)

Values are expressed as *n* (%) or median (IQR).

Abbreviations: BSA, body surface area; CPA, cardiopulmonary arrest; CPR, cardiopulmonary resuscitation; ECMO, extracorporeal membrane oxygenation; ECPR, extracorporeal cardiopulmonary resuscitation; HLHS, hypoplastic left heart syndrome; LOS, low output syndrome; TAPVC, total anomalous pulmonary venous connection; TGA, transposition of great arteries.

In the study cohort, 54 (92%) patients had congenital heart disease. Single ventricular physiology was present in 38 (64%) patients, including 20 (34%) with hypoplastic left heart syndrome or its variant and 6 (10%) with asplenia. Indications for ECMO were extracorporeal CPR in 40 (68%) patients, cardiogenic shock in 12 (20%), septic shock in 1 (2%), and respiratory failure in 6 (10%). The median manual CPR time was 36 (IQR 23-51) min.

### ECMO procedure

ECMO cannulation was performed in the intensive care unit in 57 patients and in the cardiac catheterization laboratory in 2 patients. Arterial and venous cannulas were placed directly in the aorta or innominate artery and atrium, with the chest left open, in 39 (67%) patients who underwent open-heart surgery during the same hospitalization. All patients who underwent central cannulation had previously undergone cardiac surgery through a median sternotomy. In 20 patients (33%), veno-arterial ECMO was established via cervical cannulation using the carotid artery and internal jugular vein because central cannulation was considered difficult due to postoperative adhesions. In 4 (7%) patients, a double lumen catheter was placed in the atrium under open-chest conditions or in the internal jugular vein for veno-venous ECMO. The total median ECMO duration was 4 (IQR 2-8) days.

Surgical or catheter interventions during ECMO support were summarized in **Table S2**. Interventions were performed in 23 (39%) patients and mainly consisted of procedures to correct residual or reversible cardiovascular lesions, including pulmonary flow regulation, palliative cardiac surgery, and catheter-based interventions such as ablation and balloon atrial septostomy.

### In-hospital outcomes after ECMO support

**Figure 1.**
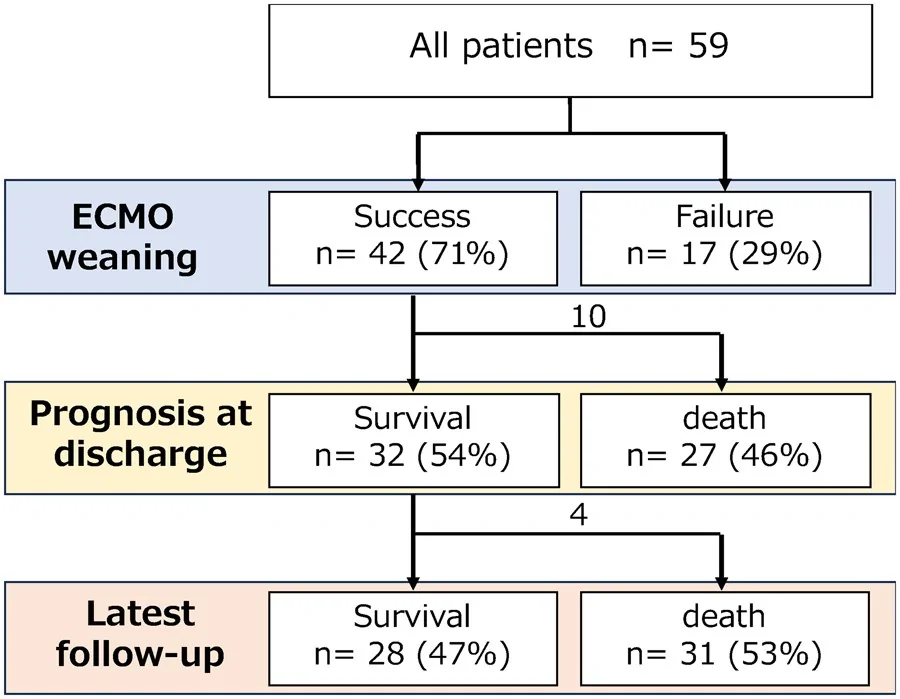
Flow Chart of Outcomes After ECMO Support. Abbreviation: ECMO, extracorporeal membrane oxygenation.

The post-ECMO outcomes are summarized in **Figure 1**. Of the 59 patients, 42 (71%) were successfully weaned from ECMO, and 32 (54%) survived until hospital discharge.

Complications during ECMO support are summarized in **Table 2**. Cerebral infarction and Haemorrhage occurred in 3 and 8 patients, respectively. In contrast, non-cerebral bleeding occurred in 8 patients, including lung/airway bleeding in 8 and intestinal bleeding in 1, with overlap in 1 case. Hypoxic encephalopathy developed in 12 (20%) patients. All intracranial complications were managed conservatively. Renal replacement therapy, such as peritoneal dialysis or extracorporeal ultrafiltration, was required in 18 (31%) patients.

**Table 2.** Complications During ECMO Support

Complication	Incidence (*n* = 59)
Stroke	3 (5)
Haemorrhage	8 (14)
Non-cerebral bleeding	8 (14)
Renal failure	18 (31)
Infection	9 (15)
Hypoxic ischaemic encephalopathy	12 (20)

Values are expressed as *n* (%).

Overall, 17 (29%) patients died during ECMO support (**Table S3**). The primary cause of death (*n* = 12) was heart failure, encompassing ventricular dysfunction in 9 patients and coronary issues in 3. The remaining 5 patients died due to respiratory failure (*n* = 2) and sepsis (*n* = 3).

Among the 42 patients successfully weaned from ECMO, 10 subsequently died during the same hospitalization; the median duration from ECMO weaning to death was 19 (IQR 15-36) days. The causes of death after ECMO weaning included ventricular dysfunction in 3 patients, sepsis in 3, respiratory failure in 1, and other causes in 3.

### Long-term survival after ECMO support

**Figure 2.**
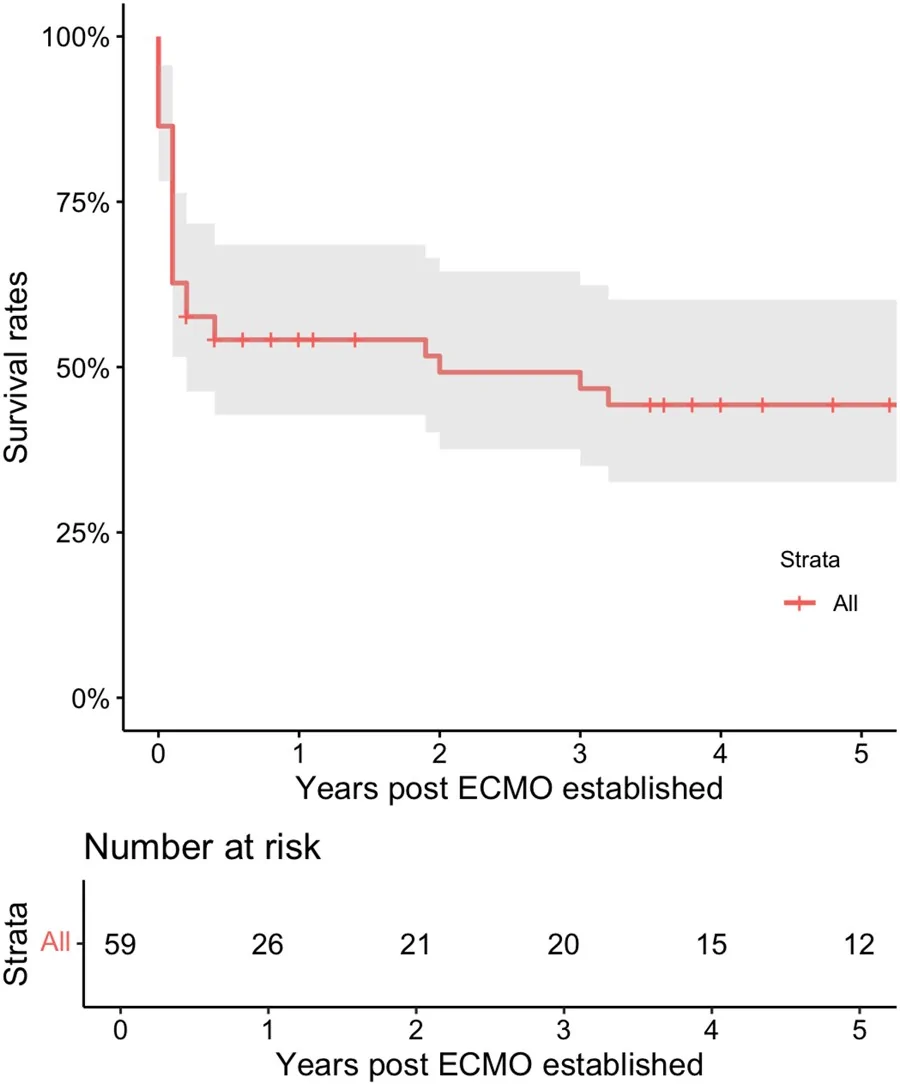
Kaplan–Meier Curve for Overall Survival After ECMO Support. Abbreviation: ECMO, extracorporeal membrane oxygenation.

The median follow-up period of hospital survivors was 3.7 (IQR 1.0-6.4) years. The overall survival rate after ECMO support was 57%, 47%, and 44% at 1, 3, and 5 years, respectively (**Figure 2**). During follow-up after hospital discharge, 4 patients had died. The causes of late death included ventricular dysfunction (*n* = 1), respiratory failure (*n* = 1), sepsis (*n* = 1), and sudden death (*n* = 1). No patients required transition to VAD support or underwent heart transplantation during follow-up after ECMO support.

### Mortality risk factors

The results of the Cox regression analysis of the factors associated with overall mortality are presented in **Table 3**. In multivariable analysis, 2 independent factors associated with mortality were identified: catheter/surgical intervention during ECMO (HR, 0.29; 95% CI, 0.12-0.67; *P* = .004) and non-cerebral bleeding (HR, 2.94; 95% CI, 1.01-8.58; *P* = .049).

**Table 3.** Multivariable Cox Regression Analysis for Mortality

	Univariable analysis			Multivariable analysis		
	HR	CI	*P*-value	HR	CI	*P*-value
Age, months	0.914	0.802-1.042	.177			
<1 month	1.946	0.941-4.026	.073			
Body weight, kg	0.709	0.480-1.047	.084			
Female sex	0.889	0.436-1.815	.747			
Single ventricle	0.653	0.324-1.315	.238			
Asplenia	1.302	0.455-3.726	.623			
Fundamental diagnosis						
Congenital heart disease	0.665	0.202-2.190	.502			
HLHS/HLHS variant	0.964	0.444-2.095	.927			
Postoperative cardiac surgery	3.375	1.022-11.148	.046			
Cause of ECMO						
ECPR	1.013	0.477-2.154	.972			
Cardiogenic shock	1.179	0.506-2.745	.702			
Sepsis	2.532	0.339-18.92	.365			
Respiratory	0.536	0.128-2.249	.394			
ECMO procedure						
ECMO duration, days	1.039	1.015-1.064	.001	1.02	0.991-1.051	.181
Location						
Intensive care unit	0.279	0.064-1.212	.089			
Cardiac catheterization laboratory	3.585	0.825-15.58	.089			
Cannulation site						
Central	1.237	0.569-2.689	.592			
Cervical	0.809	0.372-1.758	.592			
System						
Veno-arterial	1.129	0.269-4.376	.869			
Veno-venous	0.886	0.211-3.718	.869			
Post-ECMO intervention	0.277	0.119-0.649	.003	0.285	0.121-0.672	.004
Complication						
Stroke	0.473	0.064-3.475	.462			
Haemorrhage	1.399	0.534-3.663	.495			
Non-cerebral bleeding	4.111	1.694-9.98	.002	2.938	1.006-8.581	.049
Renal failure	2.541	1.23-5.249	.012			
Infection	2.32	1.024-5.255	.044			
Hypoxic encephalopathy	0.635	0.243-1.656	.353			
Secondary ECMO	2.843	1.233-6.514	.014			

Multivariable Cox regression analysis for mortality. Variables with a univariable *P*-value of <.01 were included in the multivariable analysis. HRs and 95% CIs were calculated for each variable.

Abbreviations: BSA, body surface area; CI, confidence interval; CPA, cardiopulmonary arrest; CPR, cardiopulmonary resuscitation; ECMO, extracorporeal membrane oxygenation; ECPR, extracorporeal cardiopulmonary resuscitation; HLHS, hypoplastic left heart syndrome; HR, hazard ratio; LOS, low output syndrome.

### Analysis of patients receiving CPR

**Figure 3.**
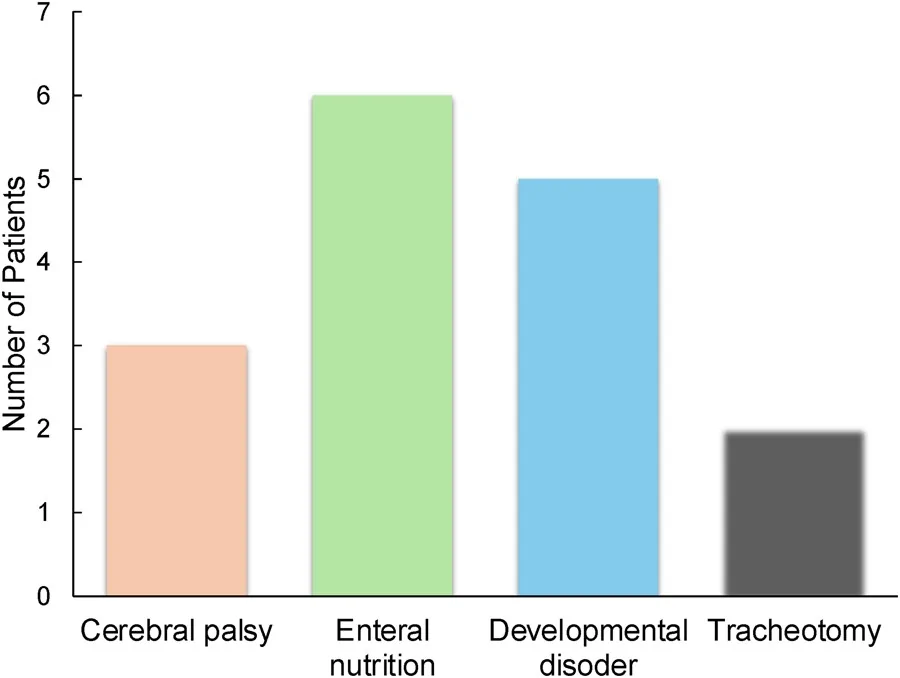
The Complications Observed in Survivors With Hypoxic Ischaemic Encephalopathy.

The baseline characteristics of patients who required CPR before ECMO support are presented in **Table 4**. In total, 40 (68%) patients required CPR, and they were divided into 2 groups based on the median CPR duration (>36 and ≤36 min). There was no significant difference in in-hospital mortality between the 2 groups (*P* = .34). In contrast, patients with a CPR duration exceeding 36 min were more likely to undergo ECMO cannulation via the cervical site (15% vs 65%, *P* = .001) and to develop HIE (5% vs 40%, *P* = .008) compared to those with a CPR duration of ≤36 min. Of the patients who received CPR, 9 subsequently developed HIE; of these, 7 survived until hospital discharge. The median follow-up period after hospital discharge was 1.1 (IQR 0.5-5.1) years. The complications observed in survivors with HIE are shown in **Figure 3**. At the latest follow-up, 5 patients were alive, 5 exhibited developmental disorders, and 3 had been diagnosed with cerebral palsy.

**Table 4.** Baseline Characteristics of Patients Requiring CPR before ECMO Support

Variable	All (*n* = 40)	CPR time ≤36 min (*n* = 20)	CPR time >36 min (*n* = 20)	*P*-value
Age <1 month	10 (25)	6 (30)	4 (20)	.465
BSA, m^2^	0.20 (0.18-0.23)	0.19 (0.17-0.22)	0.21 (0.18-0.24)	.091
Female sex	20 (50)	8 (40)	12 (60)	.206
Single ventricle	29 (73)	14 (70)	15 (75)	.723
Asplenia	3 (7)	1 (5)	2 (10)	.548
Fundamental diagnosis				
Congenital heart disease	37 (93)	18 (30)	19 (20)	.546
HLHS/HLHS variant	10 (25)	7 (35)	3 (15)	.144
Postoperative cardiac surgery	32 (80)	17 (85)	15 (75)	.429
ECMO procedure				
ECMO duration, days	4 (2-8)	5 (2-12)	6 (1-12)	.341
Cannulation site				
Cervical	16 (40)	3 (15)	13 (65)	.001
Hospital death	17 (43)	10 (50)	7 (35)	.337
Complications				
Stroke	3 (8)	2 (10)	1 (5)	.548
Haemorrhage	5 (13)	1 (5)	4 (20)	.151
Non-cerebral bleeding	5 (13)	4 (20)	1 (5)	.151
Renal failure	13 (33)	8 (40)	5 (25)	.311
Infection	5 (13)	4 (20)	1 (5)	.151
Hypoxic encephalopathy	9 (23)	1 (5)	8 (40)	.008

Values are expressed as *n* (%) or median (IQR).

Abbreviations: BSA, body surface area; CPR, cardiopulmonary resuscitation; ECMO, extracorporeal membrane oxygenation; HLHS, hypoplastic left heart syndrome; LOS, low output syndrome.

## DISCUSSION

This retrospective study indicated that survival rates in neonates and infants requiring emergent ECMO support outside the operating room were comparable to those in previous studies. Moreover, non-cerebral bleeding and catheter/surgical interventions during ECMO were identified as factors associated with mortality, while prolonged CPR duration (exceeding 36 min) was associated with HIE development.

In the present cohort of neonates and infants who required emergent ECMO support outside the operating room, the ECMO weaning rate was 71% and the survival-to-hospital discharge rate was 54%. Despite ECMO initiation outside the operating room having been associated with poor prognostic outcomes in neonates and infants,^10^ the ECMO weaning and survival-to-hospital discharge rates identified in the current study seem to be comparable or superior to those previously reported in paediatric ECMO.^1^^,^^2^^,^^5^^,^^9^^,^^11–13^

In the present study, surgical/catheter intervention was independently associated with improved survival. Previous studies have similarly reported that early detection of residual or correctable cardiovascular lesions followed by timely intervention predicted survival after successful weaning from ECMO.^10^^,^^14^^,^^15^ These findings suggest that early identification and timely correction of residual or reversible lesions remain essential determinants of successful ECMO support.

Our findings suggest that minimizing CPR duration and preventing bleeding complications may be key strategies for improving long-term outcomes in neonates and infants requiring emergent ECMO support. Several reports have suggested that prolonged CPR duration is negatively associated with mortality^7^^,^^16^^,^^17^ and may lead to poor neurological outcomes. In the current study, although CPR duration was not directly associated with mortality, CPR duration exceeding 36 min was significantly associated with HIE development. These findings are consistent with those of previous reports suggesting that achieving ECMO circulation within 30-40 min of CPR duration is critical for preserving neurologic function.^1^^,^^18–21^ Therefore, strategies aimed at minimizing CPR duration may improve long-term neurological outcomes.

In the present study, CPR duration was sometimes prolonged due to the difficulty of ECMO cannulation in neonates and infants. Patients with a CPR duration exceeding 36 min were more likely to undergo ECMO cannulation via the cervical site compared to those with a CPR duration of ≤36 min. Previous studies suggested that cervical cannulation may be technically challenging in neonates and infants during ongoing CPR because of their small body size or the presence of a cervical haematoma related to a central venous catheter.^22^^,^^23^ Therefore, rapid establishment of ECMO is key to minimizing the CPR duration. In patients considered to be at high risk of imminent circulatory collapse, early preparation for cervical cannulation, including pre-emptive exposure and vessel looping of the carotid artery and internal jugular vein, may facilitate rapid ECMO deployment and shorten CPR duration. To further shorten CPR duration, our institution has implemented a low-priming-volume ECMO system (99 ml) as part of our strategy to facilitate rapid ECMO deployment and minimize CPR duration.^24–26^

Bleeding remains a significant complication of ECMO support. Previous studies have reported that bleeding rates reached 45%-50% during ECMO and were strongly associated with mortality.^16^^,^^27^^,^^28^ In the current study, non-cerebral bleeding during ECMO was a risk factor for mortality. Moreover, neonates and infants are particularly susceptible to bleeding complications because of their immature coagulation system and the relatively large priming volume of the ECMO circuit.^29–31^ Therefore, meticulous anticoagulation management and comprehensive coagulation monitoring are essential to minimize bleeding risk. Because thromboelastography provides a global assessment of haemostatic function,^29^^,^^30^^,^^32^^,^^33^ its use in conjunction with conventional ACT and APTT monitoring may facilitate more individualized anticoagulation management during ECMO support.

The management of patients who develop HIE after ECMO remains ethically challenging. In neonates and infants, long-term neurological outcomes are often difficult to predict accurately, even in the presence of HIE. Therefore, decisions regarding continuation or limitation of medical treatment should not be based solely on the presence of HIE. Instead, treatment strategies should be determined through multidisciplinary discussion involving paediatric cardiac surgeons, intensivists, paediatricians, and allied healthcare professionals, together with shared decision-making with the patient’s family, taking into account the overall clinical condition, anticipated prognosis, and goals of care.

This study had certain limitations. First, the sample size was relatively small, which may have limited the statistical power of the study and increased the risk of a Type II error. Second, this was a non-randomized, retrospective, single-centre study. Therefore, there is an inherent risk of selection bias and residual confounding that may have influenced the results. Third, the study population was heterogeneous and included patients with different underlying diagnoses and indications for ECMO support, which may limit the generalizability of our findings. Fourth, the follow-up period was relatively short among survivors who required CPR and subsequently developed HIE, which may have been insufficient to accurately assess long-term quality of life and neurological disability. Additionally, neurological outcomes were not evaluated using standardized neurodevelopmental assessment tools. Therefore, the long-term neurological outcomes may have been under- or overestimated. Finally, because of the relatively small sample size and limited number of events, only a small number of variables could be included in the multivariable model to avoid overfitting. Therefore, potentially relevant variables, including the requirement for renal replacement therapy, were not incorporated into the final model and may have influenced the observed associations.

## CONCLUSION

The survival rate of neonates and infants who received ECMO outside of the operating room was acceptable. To further improve outcomes, effective strategies are needed to prevent bleeding complications and reduce the resuscitation time.

## Supplementary Material

ivag235_Supplementary_Data

## Data Availability

The authors declare that all data in this article are available within the article.

## Abbreviations

CPR: cardiopulmonary resuscitation
ECMO: extracorporeal membrane oxygenation
