# Supplementary material for: Extracorporeal Membrane Oxygenation Outside the Operating Room in Neonates and Infants
Source: Interdiscip Cardiovasc Thorac Surg. 2026 Aug 25;41(9):ivag235. doi: 10.1093/icvts/ivag235 (PMC13576417; doi:10.1093/icvts/ivag235)
Supplement: Supplementary file 1 [file ivag235_supplementary_data.docx]

Supplementary Table S1. Variables of missing data

| **Variable** | **Missing** |
| --- | --- |
| Body surface area | 1 (1.7) |
| Cardiopulmonary resuscitation time | 2 (3.4) |

Values are expressed as n (%). All other study variables had complete data.

Supplementary Table S2. Intervention on ECMO

| **Intervention on ECMO** | **All (n=23)** |
| --- | --- |
| Surgical operation |  |
| Shunt operation | 8 (35) |
| Additional | 3 (13) |
| Revision | 3 (13) |
| Clipping | 2 (9) |
| Valve replacement | 2 (9) |
| PA banding | 3 (13) |
| Coronary artery plasty | 1 (4) |
| RV-PA conduit revision | 1 (4) |
| IPAS | 1 (4) |
| One and half operation | 1 (4) |
| Starns | 1 (4) |
| Catheter |  |
| Ablation | 2 (9) |
| BAS | 1 (4) |
| PTA for BT shunt | 1 (4) |
| Thrombectomy for RV-PA conduit | 1 (4) |

Values are expressed as n (%)

Supplementary Table S3. Cause of death during ECMO support and hospital stay

| **Cause of death** | **All (n=27)** |
| --- | --- |
| *Weaning failure (n=17)* |  |
| Ventricular dysfunction | 9 (53) |
| Coronary trouble | 3 (18) |
| Obstruction | 2 (11) |
| Sinusoidal communication | 1 (6) |
| Respiratory | 2 (11) |
| Sepsis | 3 (18) |
| *In hospital (n=10)* |  |
| Ventricular dysfunction | 3 (33) |
| Respiratory | 1 (11) |
| Sepsis | 3 (33) |
| Others | 3 (33) |

Values are expressed as n (%)

Supplementary Appendix. Definitions of clinical variables

・neonates and infants: neonates were defined as patients aged <28 days and infants as patients aged <1 year.

・CPR duration: CPR duration was defined as the time interval from initiation of manual chest compression to establishment of ECMO.

・Successful ECMO weaning: Successful ECMO weaning was defined as survival for more than 24 hours after ECMO decannulation.

・Hypoxic encephalopathy: Hypoxic encephalopathy was diagnosed based on clinical neurological findings and computed tomography findings interpreted by radiologists.

・Infection: Infection was defined as clinically suspected infection with positive blood culture results during ECMO support.

・Non-cerebral bleeding: Non-cerebral bleeding was defined as any bleeding occurring outside the central nervous system.
